# Psychological Flexibility and Proactive Career Behaviors During the University to Work Transition: A Longitudinal Analysis

**DOI:** 10.1002/pchj.70083

**Published:** 2026-03-04

**Authors:** Hacı Arif Doğanülkü, Sıdıka Ece Yılmaz

**Affiliations:** ^1^ Department of Guidance and Psychological Counseling/Educational Sciences, Çukurova University Adana Türkiye; ^2^ Economics, Administrative and Social Sciences/Tourism Management Adana Alparslan Türkeş Science and Technology University Adana Türkiye

**Keywords:** career adaptability, career development, cross‐lagged panel model, longitudinal mediation, proactive career behaviors, psychological flexibility

## Abstract

Proactive career behaviors facilitate the transition from student to employee, and understanding the mechanisms behind their development is crucial for informing strategies that promote these behaviors. The literature indicates that the development of proactive career behaviors has been investigated in several studies; however, most of these studies employed a cross‐sectional research methodology, thereby limiting the ability to draw robust causal inferences. This study is to investigate the mediating function of students' career adaptability in the relationship between psychological flexibility and proactive career behaviors throughout a longitudinal framework. The research involved 310 Turkish university students using convenience sampling. A two‐wave cross‐lagged panel model was tested within a structural equation modeling framework to examine cross‐lagged effect among the variables over time. The findings indicated that students' career adaptability significantly mediated the longitudinal relationship between proactive career behaviors and psychological flexibility. It shows that interventions designed to enhance psychological flexibility and career adaptation within career counseling services can effectively boost students' proactive career behaviors. When assessed by human resources specialists, graduates with high psychological flexibility and career adaptability may exhibit more proactive and adaptable career behaviors, emphasizing the importance of including these attributes in recruitment processes.

## 1 Introduction

The university phase represents the final stage before entering the workforce. It signifies a transitional stage in career development processes. The career‐related activities individuals engage in during this phase significantly facilitate their smooth transition to professional life post‐graduation (Baluku et al. 2021; Saks 2018). But globalization and rising job insecurity have left today's labor markets in a situation of unprecedented unpredictability (Baluku et al. 2021). This challenging transition has converted university education from a purely academic endeavor into a vital struggle for survival in the shift to professional life. Also, the opportunities and challenges that students face after graduation are further shaped by broader labor market trends as they negotiate this crucial stage. Global data reveal that substantial issues remain concerning young employment. Young unemployment reached 13% in 2023, and the International Labour Organization's (ILO 2024) report underscores the persistent uncertainty in this field. Acquiring employment has become progressively challenging for university graduates. This underscores the need to utilize psychological and behavioral resources to ensure the smooth integration of graduates into the labor market (ILO 2024).

Considering this situation, studies assert that navigating this uncertainty and the challenging transition process requires individuals to actively manage their careers instead of remaining passive (Blokker et al. 2023; Schoon 2020). By supporting this approach, proactive career behaviors emerge as a crucial strategy, characterized by acts undertaken by individuals independently to attain their career objectives (De Weerdt et al. 2024; Hirschi et al. 2015; Wilhelm et al. 2023; Zhang et al. 2023). Examining proactive career behaviors among university students is crucial, as such behaviors have been shown to help individuals meet career‐related demands and seek more suitable employment opportunities (Yu and Davis 2016).

Proactive career behaviors serve as a mechanism that facilitates the development of career competencies, enabling individuals to thrive and progress in professional environments through diverse behavioral forms (De Vos et al. 2009; Hirschi et al. 2015). Research indicates that proactive behaviors during the time of their graduation, including networking and career planning, significantly enhance career success (De Vos et al. 2009). Accordingly, university students must demonstrate proactive career behaviors to enhance their employability prospects during their university education (Bernabé et al. 2024).

The significance of proactive career behaviors is evident; nevertheless, our comprehension of the antecedents that drive these behaviors in university students remains insufficient (Peng et al. 2021; Spurk et al. 2020). Proactive career behavior has been mostly studied in relation to working life in the literature (Wang, Hou, et al. 2024). Although research on proactive career behaviors in student populations is scarce, the topic has been analyzed concerning several antecedents across different groups. Consistent with this emphasis on individual psychological resources, previous research has demonstrated the significance of personal beliefs and cognitions in influencing proactive career behaviors. Career self‐efficacy beliefs have been demonstrated to predict individuals' proactive career behaviors (Hirschi et al. 2013), while the prominence of the future work self has been shown to affect individuals' career planning and networking behaviors (Taber and Blankemeyer 2015). Moreover, family factors, such as family motivation and career goal setting (Wang, Hou, et al. 2024) or commitment to parental responsibilities (Chen et al. 2023), have been recognized as influences on proactive career behaviors. Nevertheless, most of these studies have centered on beliefs, objectives, or social factors and have inadequately examined the dynamic cognitive and emotional processes that facilitate proactive career behavior in ambiguous or shifting circumstances. Although research has offered considerable insights into motivational and social factors, the impact of an individual's capacity to smoothly navigate internal and external problems on proactive career engagement remains unclear.

Psychological flexibility has recently been discussed in the context of individuals' careers and has attracted the attention of researchers (Chong et al. 2023; Kuo et al. 2018; Puolakanaho et al. 2020). It assists individuals in making sensible decisions and formulating suitable strategies among career‐related uncertainty and possibilities. It can serve as a foundation for individuals to cultivate career adaptability abilities. For example, flexible individuals can more readily acquire the ability to strategize their careers, enhance their talents, and proactively evaluate possibilities (Savickas and Porfeli 2012). Psychological flexibility is extremely effective in fully demonstrating not only social but also professional functionality (Bonanno et al. 2004). Fixed personality traits (Dursun and Argan 2017; Rahbar Zeraati et al. 2024) have been the focus of most research in terms of forming career adaptability, while dynamic cognitive processes like psychological flexibility, which enable people to mentally adjust to changing situations, have not received enough attention. More significantly, a particular study (Storme et al. 2020) highlighted the significance of “intrapersonal variability” in understanding the career adaptability process, which indicates that personality should not be viewed as a rigid framework but rather as a structure that may change and adapt based on the individual and circumstance. In other words, it suggests that emphasis and investigation should be placed on flexibility and context‐sensitive behavioral patterns rather than set traits.

Although prior research has shown that proactive career behaviors can be explained by diverse motivational and social factors, the psychological mechanisms that mediate the emergence of these behaviors in people remain underexplored. The relationship between psychological flexibility and individuals' ability to cultivate proactive career behaviors, whether direct or indirect, has not been thoroughly investigated. Career adaptability is frequently discussed in literature as a significant predictor of proactive career behaviors; however, the influence of psychological flexibility in enhancing these adaptive capabilities has yet to be thoroughly examined (Savickas and Porfeli 2012; Hirschi 2009). This difference underscores the significance, both theoretically and empirically, of viewing career adaptability as a mediating factor in the relationship between psychological flexibility and proactive career behaviors.

Moreover, prior research on these variables has been cross‐sectional. The trajectory of these behaviors over time has not been examined. Unlike previous cross‐sectional studies, this study addresses the identified gaps by employing a longitudinal methodology. Consequently, the study's major purpose is to investigate the impact of university students' psychological flexibility on their proactive career behaviors over time by examining the role of career adaptability.

The research offers both theoretical and practical contributions. This study examines the early stages of career transition using a sample of university students. Thus, it provides immediately relevant results for policy and career guidance. Moreover, longitudinal results can provide career centers and university counselors with concrete evidence that interventions based on psychological flexibility can enhance student proactivity and subsequently, employment opportunities.

## 2 Literature Review

### 2.1 Theoretical Background

This study employs three primary theoretical frameworks that describe the interactions among psychological flexibility, career adaptability, and proactive career behaviors: Career Construction Theory (CCT), Career Construction Model of Adaptation (CCMA), and Acceptance and Commitment Theory (ACT).

**FIGURE 1:**
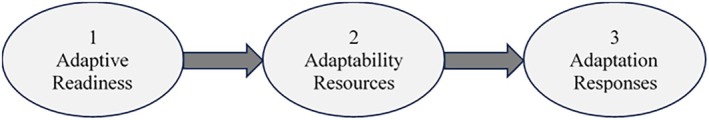
Career construction model of adaptation.

CCT argues that individuals form their careers by attributing meaning to their vocational actions and professional experiences. Individuals develop their careers by adapting to the responsibilities, transitions, and traumas they face throughout their lives. Accordingly, career development is influenced by how individuals interpret environmental demands and the psychological resources they employ in response to these demands. CCT highlights the psychological resources that affect individuals' ability to manage career‐related difficulties (Savickas 2005, 2013). Within the overarching framework of CCT, the CCMA functions as the customized theoretical lens for this study. The CCMA offers a structured framework for assessing how individuals handle career transitions and tackle difficulties with development (Savickas 2013). It explains the lifelong career building process and provides an important theoretical framework for the mediating effect of career adaptability on the relationship between individuals' psychological flexibility and proactive career behaviors. The first of the resources in CCMA is adaptive readiness, which indicates a personality trait that means being willing and ready for change. Resources are needed to make appropriate choices along with adaptive readiness. Therefore, the second element in the CCMA is adaptability resources, which refers to the psychosocial structure that indicates self‐regulation resources to cope with change. Adaptability resources are resources that address changing career circumstances and enable professional choice‐making behaviors to be fulfilled. The third dimension is adaptation responses. Adaptation responses refer to the individual's reactions to changing conditions in their career. The pictorial representation of CCMA is presented in Figure 1.

CCMA offers the process model, whereas ACT supplies the theoretical foundation for the independent variable, psychological flexibility. ACT is a theoretical approach that was developed as one of the cognitive behavioral therapies and focuses on the role of experiential learning and psychological processes in explaining human behavior (Hayes et al. 1999). The theory aims to enable individuals to participate in significant and intentional behaviors aligned with their values while managing adverse or inevitable circumstances in their personal lives. It states that behavior is influenced by both external conditions and the individual's engagement with their internal experiences, thoughts, and emotions. It offers a robust theoretical framework for comprehending the impact of psychological processes on individual behavior (Hayes et al. 2006). Hayes et al. (2006) assert that psychological flexibility, that is, the ability to remain present and behave in accordance with one's values, is a crucial element of effective functioning.

The study integrates these theories by aligning the research variables with the CCMA sequence to fulfill the research objectives. According to ACT, psychological flexibility is characterized as the adaptive readiness within the CCMA framework. It represents the essential psychological state that prepares the individual for professional responsibilities. In accordance with CCT, career adaptability denotes the resources for adaptability that an individual uses based on their readiness. Ultimately, proactive career behaviors indicate adaptive responses. The CCMA states that adaptive readiness is inadequate for generating adaptation responses. First, it must engage with adaptability resources (Savickas 2013). Thus, the theory argues that career adaptability functions as a key driving mechanism that converts psychological flexibility into proactive career behaviors.

### 2.2 Psychological Flexibility and Proactive Career Behaviors

Proactive career behaviors refer to the self‐directed actions taken to reach career aims (De Vos et al. 2009). In other words, these behaviors represent the behaviors that individuals take an action autonomously, rather than behaviors that are done at the request of someone else or with a sense of duty. Exploratory activities and opportunity seeking are among the most basic proactive career behaviors (De Vos et al. 2009; Sonnentag 2017). These behaviors reflect the responsibilities of individuals to foresee opportunities in their career lives and overcome the risks they may encounter (De Vos and Soens 2008; Neneh 2019). Proactive career behaviors most broadly describe networking, career exploration, developing specific plans, goal setting, participating in skill development activities, and mentor support (Claes and Ruiz‐Quintanilla 1998; Sonnentag 2017). Networking involves creating connections with new individuals. Career exploration involves identifying opportunities. Formulating detailed plans serves as a navigational tool to attain objectives. Goal setting involves determining a target to achieve. Engaging in skill development activities underscores the importance of continual relevance and receptiveness to growth. Ultimately, mentor support entails gaining advantages from experienced individuals. These constitute the most significant proactive career behaviors. Proactive career behaviors are associated with future work self‐concept (Bernabé et al. 2024). Additionally, these behaviors help individuals cope with career problems and seize opportunities to achieve their career aims (Jiang et al. 2023). Participating in proactive career behaviors during the transition from education to employment fosters favorable outcomes in individuals' professional success (De Vos et al. 2009; Smale et al. 2019). Consequently, it is underscored that implementing frameworks that foster proactive career behaviors is essential for both career counseling practitioners and individuals engaged in the transition of young graduates to professional life (De Vos et al. 2009).

A reduction in psychological flexibility may hinder behavioral regulation and impede the manifestation of goal‐directed behavior (Hayes et al. 2011; Masuda and Tully 2012). This situation may distract individuals from engaging in proactive career behaviors. Psychological flexibility is one of the important constructs of ACT, which is defined as the third generation and accepted as one of the cognitive‐behavioral therapies. In the context of ACT, psychological flexibility is an important trait for mental health (Karataş and Selçuk 2023). Psychological flexibility refers to an individual's ability to react to evolving environmental demands by modifying perspectives and behaviors, efficiently reallocating cognitive resources, and sustaining harmony among conflicting requirements and life domains. This adaptive capability is maintaining openness to and awareness of current events, including challenging emotions and ideas, while participating in actions aligned with one's values and goal‐directed actions. Psychological flexibility consists of six fundamental processes, which are acceptance (present‐moment awareness), self‐as‐context (cognitive defusion), values, and committed action. They collectively serve as helpful psychological skills, allowing individuals to respond adaptively rather than inflexibly to internal and external pressures, thus enhancing psychological well‐being (Bond et al. 2013; Hayes et al. 2011; Kashdan and Jonathan 2010).

Additionally, psychological flexibility is emphasized as a precondition for healthy development (Bohlmeijer et al. 2015; Fledderus et al. 2013). This might also be valid for individuals' career development. Kuo et al. (2018) state that psychological flexibility offers a comparatively effective way to promote proactive behaviors. On the contrary, low levels of psychological flexibility are characterized by difficulty in regulating behavior and an inability to exhibit goal‐oriented behaviors (Hayes et al. 2011; Masuda and Tully 2012). This situation may cause individuals to exhibit a reactive attitude that is opposite to their proactive career behavior.

Being proactive entails actively pursuing and capitalizing on opportunities to accomplish objectives (Cangiano and Parker 2015; Parker et al. 2010). On the other side, psychological flexibility is a competence that helps identify and evaluate opportunities in current conditions (Bond et al. 2008). Due to this situation, increased psychological flexibility may be the source of a rise in individuals' proactive career behaviors. In a cross‐sectional study conducted with working individuals consistent with these statements, psychological flexibility was found to be a predictor of proactive behaviors (Kuo et al. 2018). The hypothesis developed for testing in this context is presented below:

*Psychological flexibility predicts proactive career behaviors*.

### 2.3 Career Adaptability as a Mediator

Career adaptability is one of the most important concepts of CCT (Savickas 1997, 2005, 2013). Career adaptability has been defined as the state of readiness to cope with predictable tasks, such as preparing for and participating in a job role, and unpredictable situations caused by changes in employment and working conditions (Savickas 1997). Savickas (1997) states that career adaptability has four different dimensions: (1) concern, which refers to concern about one's future; (2) confidence, which refers to the belief that one can achieve one's goals; (3) curiosity about the professional world; (4) control, that is, awareness that the future is partially controllable. These dimensions express career adaptation as a whole and are one of the important psychosocial resources in the individual's career development process (Savickas 1997, 2005).

In the CCMA context, psychological flexibility can be considered as adaptive readiness (Hayes et al. 2011; Masuda and Tully 2012). Because psychological flexibility has the power to activate self‐regulation and makes the individual ready for adaptation (Waldeck et al. 2021). As a matter of fact, Hayes et al. (1996) suggest that psychological flexibility is the ability to adjust to changes in environmental conditions. On the other hand, it is also emphasized that low psychological flexibility makes it difficult to adapt to challenging situations or contexts (Waldeck et al. 2021). This situation is suitable for transforming psychological flexibility into adaptive readiness (Savickas 2013). Career adaptability is the second step in the model, namely, adaptability resources. Since individuals with psychological flexibility are highly likely to activate their adaptation resources (Hayes et al. 2011; Masuda and Tully 2012; Waldeck et al. 2021). That is, psychological flexibility functions as a feeder for adaptation. Individuals engage in proactive career behaviors at the end of their career adaptability skills (Hirschi et al. 2015; Savickas 2005). In other words, proactive career behaviors are considered an adaptation response, and individuals use these behaviors to accomplish career development tasks (Guan et al. 2017; Hirschi et al. 2015; Savickas 2005). As a matter of fact, Wang, Li, et al. (2024) emphasize that career adaptability is one of the sources that provide the main driving force for individuals' participation in proactive career behaviors. In summary, within the CCMA framework, the initial stage, psychological flexibility, signifies adaptable readiness; the subsequent phase, career adaptation, denotes adaptability resources; and the final step, proactive career behaviors, embodies adaptation responses. In this context, career adaptability provides as an important mediator between psychological flexibility and proactive career behaviors. Consequently, the study argues that adaptive readiness (psychological flexibility) necessitates the activation of adaptability resources (career adaptability) to promote adaptive responses (proactive career behaviors). To empirically test this theoretical view, the following hypothesis was developed:

*Career adaptability has a mediating effect in the relationship between psychological flexibility and proactive career behaviors*.

**FIGURE 2:**
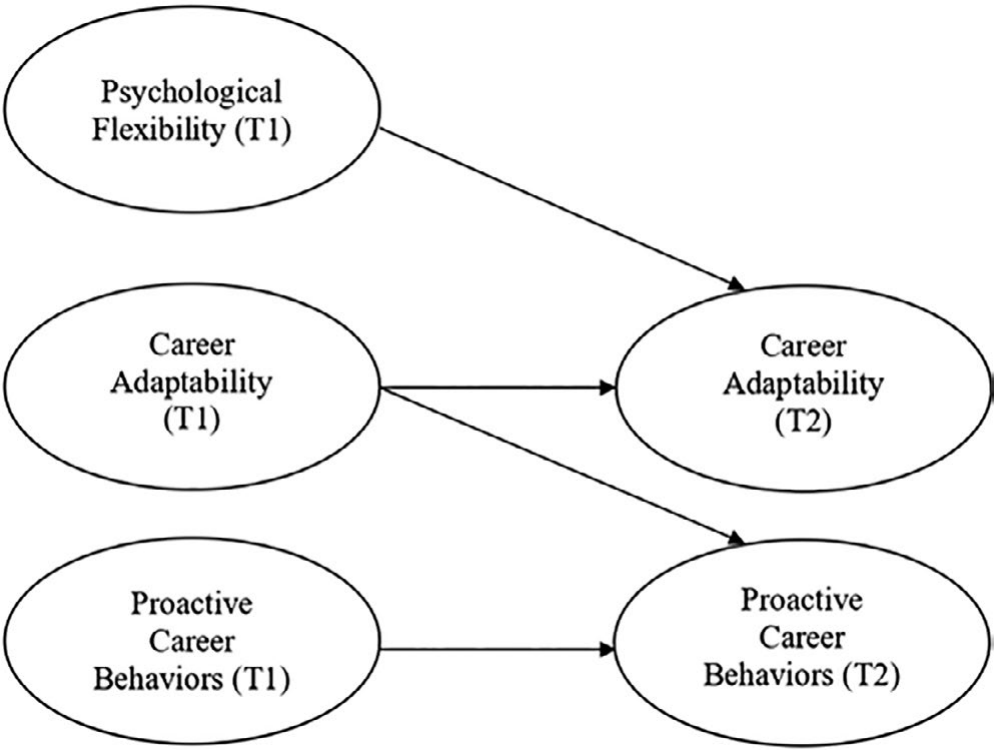
Research model.

The outcomes of this study have the capacity to ensure important contributions to the improvement of proactive career behaviors of university students. Additionally, the critical nature of identifying predictors of these behaviors requires longitudinal studies. Longer‐term longitudinal studies may provide evidence that will more clearly demonstrate the mechanism of proactive career behaviors. The hypothetical model of the research is presented in Figure 2.

## 3 Method

### 3.1 Participant and Procedure

Ethical approval for this study was obtained from the Ethical Compliance Review Board for Scientific Research of Çukurova University (30.11.2022‐E.569391). Following this approval, the data collection process commenced. The participants of the study were comprised of undergraduate students from Çukurova University, a prominent and well‐established public university located in the Mediterranean region of Türkiye. The chosen university draws a heterogeneous student population from multiple geographical areas and socio‐economic levels nationwide. This diversity improves the sample's representativeness concerning the wider university student population in Türkiye. Additionally, this sample is especially appropriate for the purposes of the study, as the transition from university to professional life has become increasingly difficult for young adults in Türkiye. Recent national statistics indicate that university graduates require an average of 13.9 months to secure employment (TURKSTAT 2023), highlighting the uncertainties and challenges they face during this period.

Convenience sampling was used as the sampling method. The method was selected due to its provision of rapid and direct access to the university students who comprise the study's target group. To enhance the representativeness of the findings, the survey was conducted with students from several faculties and academic disciplines.

The research announcement was disseminated throughout the university's several faculties through online classroom systems. Data was gathered online by distributing a link to the survey to students who consented to participate. The initial page of the survey contained details regarding the study's objective, confidentiality protocols, and the rights of participants. Participants who selected the consent option were incorporated into the study. Participant data were obtained at two distinct time points. The initial measurement (Time 1) occurred at the commencement of 2023 (January), and the subsequent measurement (Time 2) transpired 1 month later. Participants were instructed to utilize the identical code during both data gathering phases to guarantee data congruence. The mean completion time varied between 10 and 15 min. The entire process was executed in compliance with confidentiality rules.

Participants were chosen based on particular criteria. The fundamental inclusion criteria comprised possessing an undergraduate degree from the state university where the research was executed, engaging in volunteer activities, and fully completing the questionnaires at both data collection intervals (Time 1 and Time 2). Additionally, the absence of participation in a career counseling process was included as a criterion for admission. This criterion assumes that career counseling experience may affect participants' proactive career behaviors and adaptability. Participants lacking complete data forms or those whose matches could not be aligned at both time points were omitted from the study. Three hundred and twenty‐four (female: 188; male: 136) university students participated in the first data collection phase (Time 1). Three hundred and ten (female: 184; male: 126) university students participated in the second data collection phase (Time 2). At Time 2, the 14 students present at Time 1 were absent. After matching at the end of the two waves, three hundred and ten participants (female: 184; male: 126) formed the end sample of the research.

### 3.2 Measures

#### 3.2.1 Psychological Flexibility Scale

The scale was developed by Uygur and Karaca (2020) in accordance with Turkish culture and university students. There are 16 items on the scale. This scale has five sub‐dimensions in total and is a five‐point Likert type. When individuals get high scores from the scale, it shows that they have more psychological flexibility. Cronbach's alpha coefficient for the total score of the scale calculated during the development phase is 0.83. The findings of the statistical analyses show that the goodness of fit values are at an adequate level (*χ* ^2^/sd = 2.62, RMSEA = 0.05, SRMR = 0.05, GFI = 0.95, AGFI = 0.92, CFI = 0.93, NFI = 0.90, NNFI = 0.90, IFI = 0.93).

#### 3.2.2 Career Adapt‐Abilities Scale‐Short Form

This scale is a short form of the original 24‐item Career Adaptability Scale‐International Form (Savickas and Porfeli 2012). The short form of the scale was developed by Maggiori et al. (2017) and aimed to measure individuals' career adaptability. The Turkish adaptation of the scale was performed by Işık et al. (2018). There are 12 items in the scale. This scale, which has four sub‐dimensions, is a five‐point Likert‐type scale. High scores obtained by individuals from this short form of the scale mean that their career adaptability is high. The scale adaptation process was conducted with high school students, university students, and working individuals. Cronbach's alpha values calculated within the scope of reliability analyses were found between 0.80 and 0.91 in these three groups. Confirmatory factor analyses calculated within the scope of validity analysis found the goodness of fit indices within these three groups to be in an acceptable range (*χ* ^2^/df = between 2.13 and 3.38, GFI = between 0.950 and 0.960, CFI = between 0.941 and 0.966, TLI = between 0.922 and 0.955, RMSEA = between 0.059 and 0.082).

#### 3.2.3 Career Engagement Scale

The instrument was developed to measure the level of proactive career behaviors of individuals (Hirschi et al. 2014). It was adapted into Turkish by Korkmaz et al. (2020). There are nine items in the Career Engagement Scale. This scale is in the form of a five‐point Likert, and there are no sub‐dimensions. Higher scores that individuals receive from the scale reveal that they exhibit more proactive career behaviors. Cronbach's alpha coefficient, which was checked for reliability analysis during the scale adaptation process, was found to be 0.81. Confirmatory factor analyses calculated within the scope of validity analysis found the goodness of fit indices for the single factor model like in the original scale to be in an acceptable range (*χ* ^2^/df = 4.9; RMSEA = 0.09; SRMR = 0.06; TLI = 0.93; GFI = 0.92).

### 3.3 Data Analysis

In current research, it was investigated whether career adaptability had a mediating role in the longitudinal relationship between psychological flexibility and proactive career behaviors. In preliminary analyses, correlation values, descriptive statistics (means, standard deviations, skewness, and kurtosis), and reliability coefficients of the variables were analyzed. Then, in order to obtain data regarding the variables of career adaptability, psychological flexibility, and proactive career behaviors, the data collected at two different times (Time 1 and Time 2) were examined using autoregressive analysis of the cross‐lagged panel model, which is a semi‐longitudinal relational research design (Cole and Maxwell 2003; Preacher 2015). The criteria used for testing the goodness of fit of the structural model to be formed were as follows: *χ* ^2^/df < 5, CFI > 0.90, GFI > 0.90, TLI > 0.90, and RMSEA < 0.10 (Kline 2023; Marcoulides and Schumacher 2001; Tabachnick and Fidell 2012). Data analysis was performed using SPSS 26.0 and AMOS Graphics 24.

### 3.4 Control Variables

Research indicates that males and females may differ in their demonstration of proactive behavior (Maki et al. 2005). Age has also been highlighted as a factor that can influence the desire to exhibit proactive behavior (Truxillo et al. 2012). Both age and gender have been considered as control factors in proactive behavior models in previous studies (Jiang et al. 2024; Smale et al. 2019). Consistent with these findings, age and gender were incorporated as control variables in the model of this study.

## 4 Results

### 4.1 Preliminary Analysis

Among the participants, 88 (28.4%) were enrolled in education sciences, 63 (20.3%) in engineering, 86 (27.7%) in health sciences, and 73 (23.6%) in economics and administrative sciences. Of the participants, 184 (59.4%) were female and 126 (40.6%) were male. Additionally, on perceived socioeconomic status, 59 (19%) of the individuals indicated low status, 214 (69%) reported medium status, and 37 (12%) identified as high status. This distribution aligns with the socioeconomic framework of the whole Turkish university student demographic. The age ranges between 18 and 23, and the mean of the age is 20.11 (SD = 2.57). The demographic characteristics of the participants are displayed in Table 1.

**TABLE 1:** Demographic characteristics of participants.

Variable	Category	*n*	%
Gender	Female	184	59.4%
	Male	126	40.6%
Academic fields	Education	88	28.4%
	Engineering	63	20.3%
	Health sciences	86	27.7%
	Economics and administrative sciences	73	23.6%
Perceived socioeconomic level	Low	59	19%
	Medium	214	69%
	High	37	12%

It was observed that the reliability values of McDonald's omega (*ω*), Guttman's lambda 6 (*λ*6), and Cronbach's alpha (*α*) of all variables were within appropriate values at both Time 1 and Time 2. Both skewness and kurtosis coefficients are between ±1, and this result indicates a normal distribution. Significant relationships were obtained between the variables of psychological flexibility, career adaptability, and proactive career behaviors at both Time 1 and Time 2. Also, a collinearity test was performed to evaluate common method bias, adhering to the methods by Kock (2015). All variables were regressed against one another, and the Variance Inflation Factor (VIF) values were analyzed. The values for all components were below the recommended threshold of 3.3 (range from 1.14 to 1.27), suggesting that common method bias was not a significant issue in this study. The values of descriptive statistics, correlation coefficients, and reliability coefficients for all variables in the research are shown in Table 2.

**TABLE 2:** Reliability, descriptive statistics, and correlations for the study variables.

Variable	Reliabilities and descriptive statistics							Correlations				
	*α*	*ω*	*λ*6	Mean	SD	Skewness	Kurtosis	1	2	3	4	5
1. Psychological Flexibility T1	0.81	0.82	0.88	48.69	8.68	−0.01	−0.70	—				
2. Psychological Flexibility T2	0.81	0.84	0.89	45.57	8.75	0.12	−0.69	57**	—			
3. Career Adaptability T1	0.87	0.87	0.88	37.35	8.12	0.39	−0.49	46**	31**	—		
4. Career Adaptability T2	0.86	0.86	0.87	38.81	7.18	0.54	−0.36	62**	36**	61**	—	
5. Proactive Career Behaviors T1	0.91	0.91	0.92	29.82	7.72	−0.16	−0.23	39**	47**	36**	40**	—
6. Proactive Career Behaviors T2	0.84	0.84	0.85	30.70	5.68	−0.59	0.48	41**	38**	48**	46**	69**

*Note:* ** *p* < 0.01, SD = standard deviation, *N* = 310. For reliability analyses, Cronbach's alpha (*α*), McDonald's omega (*ω*), and Guttman's lambda (*λ*6) are reported.

### 4.2 Measurement Model Analysis

Subsequent to the data collecting process, the measurement model was assessed using Confirmatory Factor Analysis (CFA), and convergent validity was established by Average Variance Extracted (AVE). Discriminant validity was evaluated by computing the Heterotrait–Monotrait (HTMT) ratio. Internal consistency reliability was evaluated using Cronbach's alpha and composite reliability (CR) utilizing the datasets acquired at both Time 1 and Time 2.

Fornell and Larcker (1981) assert that AVE values exceeding 0.50 signify that each concept is sufficiently elucidated by the corresponding items, hence confirming convergent validity. AVE values range between 0.44 and 0.62 for Time 1 and Time 2. If the AVE is slightly below the 0.50 level, it may still be deemed acceptable regarding convergent validity (Hair et al. 2019). Psychological flexibility (0.44) and proactive career behaviors (0.45) exhibited AVE values somewhat below the suggested 0.50 threshold. Convergent validity may still be deemed sufficient when the AVE is below 0.50, as long as the associated composite reliability surpasses 0.60 (Fornell and Larcker 1981). In this study, both constructs fulfilled this criterion. CR values range between 0.79 and 0.91; all values are higher than 0.60 (Fornell and Larcker 1981). The findings validate the data sets, measurement model, and pertinent scales employed in the study as reliable and effective in assessing the identified components. Hair et al. (2022) assert that standardized factor loadings exceeding 0.40 are adequate for the measurement model to satisfy the requirements for convergent validity. Results indicate that the measurement model analyses performed on the data sets collected at both Time 1 and Time 2 demonstrated elevated levels of convergent validity. The AVE and CR values and normalized factor loadings are displayed in Table 3.

**TABLE 3:** Measurement model results.

Construct	Factor loading range	CR	AVE
Time 1			
Psychological flexibility	0.43–0.75	0.79	0.44
Career adaptability	0.73–0.80	0.85	0.58
Proactive career behaviors	0.48–0.89	0.91	0.54
Time 2			
Psychological flexibility	0.45–0.77	0.84	0.51
Career adaptability	0.73–0.81	0.86	0.62
Proactive career behaviors	0.40–0.77	0.87	0.45

*Note:* All standardized factor loadings were significant (*p* < 0.001).

A key part of assessing measuring instruments is establishing that the constructs within the model are adequately distinct from one another. Ensuring discriminant validity mitigates the possibility of conceptual overlap among constructs and enhances the accuracy of result interpretation. To this end, the HTMT ratio (Henseler et al. 2015), a commonly employed technique in the literature, was computed. The results indicate that all HTMT levels are below the 0.90 criterion. Henseler et al. (2015) assert that an HTMT score under 0.90 signifies the attainment of discriminant validity. Consequently, there is no issue of discriminant validity among the conceptions. HTMT analysis results are displayed in Table 4.

**TABLE 4:** Heterotrait–Monotrait ratio (HTMT).

	1	2	3	4	5	6
1. Psychological flexibility T1	—					
2. Psychological flexibility T2	0.78	—				
3. Career adaptability T1	0.56	0.58	—			
4. Career adaptability T2	0.65	0.67	0.77	—		
5. Proactive career behaviors T1	0.46	0.52	0.55	0.51	—	
6. Proactive career behaviors T2	0.51	0.58	0.61	0.56	0.69	—

### 4.3 Longitudinal Mediational Model

**FIGURE 3:**
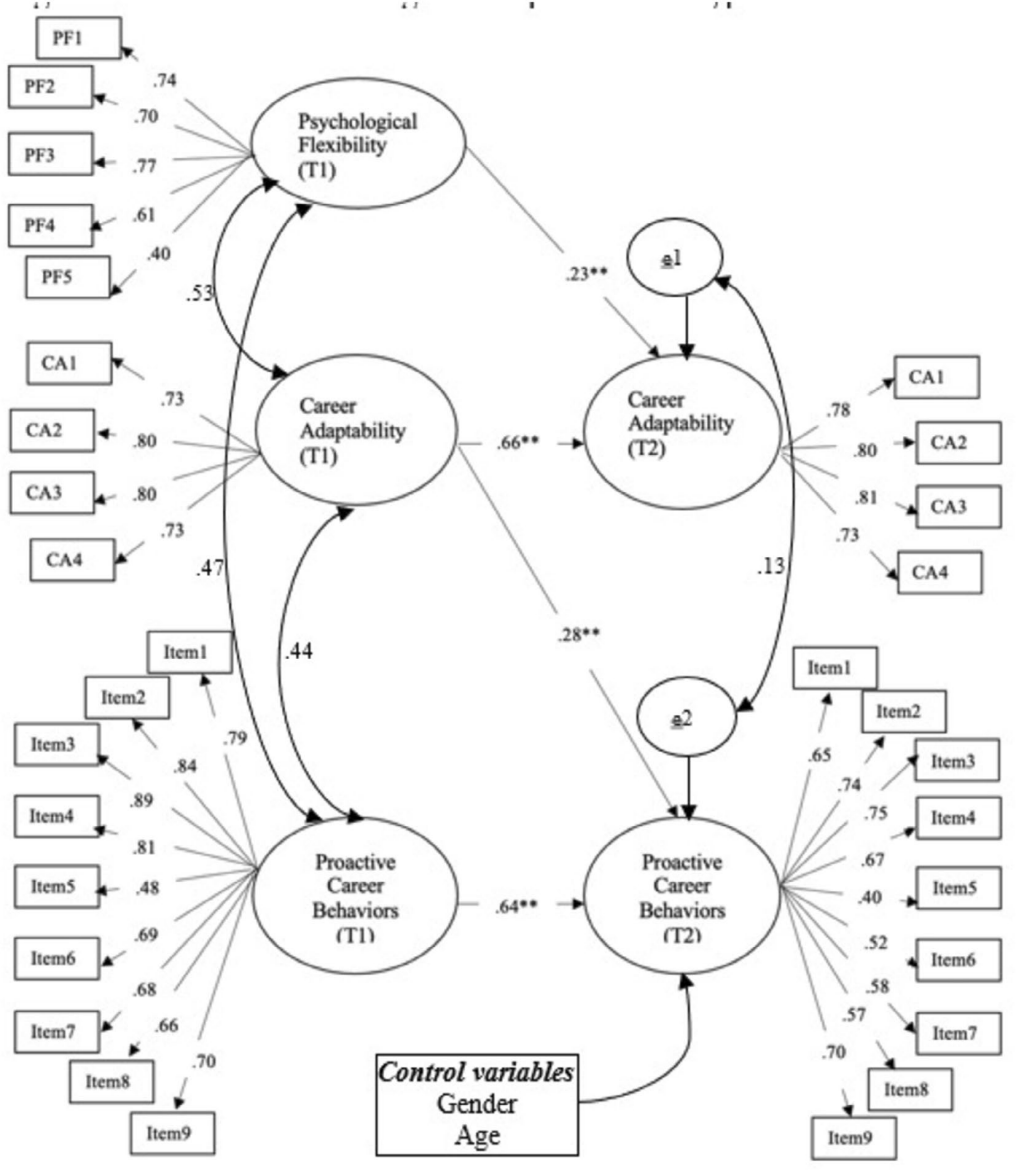
Cross‐lagged panel model for a semi‐longitudinal design for investigating the indirect association between psychological flexibility and proactive career behaviors through career adaptability (** *p* < 0.01).

A cross‐lagged panel design was selected to investigate whether career adaptability mediates the effect of psychological flexibility on proactive career behaviors in a semi‐longitudinal model (Cole and Maxwell 2003). Based on the cross‐lagged panel model for a semi‐longitudinal design, psychological flexibility at Time 1 was a significant positive predictor of career adaptability at Time 2 (controlling for career adaptability at Time 1; *β* = 0.23, SE = 0.205, 95% CI = 0.83–0.372). Also, career adaptability at Time 1 was a significant positive predictor of proactive career behaviors at Time 2 (controlling for proactive career behaviors at Time 1; *β* = 0.28, SE = 0.018, 95% CI = 0.152–0.396). The cross‐lagged panel model for a semi‐longitudinal model fits the data well: *χ* ^2^/df = 2.64, CFI = 0.93, GFI = 0.91, RMSEA = 0.71. Figure 3 demonstrates the longitudinal paths of the hypothetical model.

### 4.4 Control Variables

The analysis of control variables revealed no significant connection between proactive career behaviors and participants' gender (*β* = 0.012, *p* > 0.5) and age (*β* = 0.023, *p* > 0.5). The results of all hypotheses remained consistent even when controlling for characteristics of gender and age. The absence of confounding effects is demonstrated by the lack of substantial changes in the estimations when accounting for characteristics like gender and age.

## 5 Discussion

### 5.1 General Discussion

This study offers a novel contribution to career literature by transitioning from the primarily cross‐sectional correlations explored in current research to a longitudinal viewpoint. This research employs a cross‐lagged panel methodology, offering robust evidence for a causal direction frequently presumed but not sufficiently examined in prior studies. The findings experimentally validate a longitudinal process whereby psychological flexibility acts as a driver to enhanced career adaptability over time, thereby encouraging proactive career behaviors. This is significant as it recognizes career adaptability not merely as a correlated variable but also as a vital longitudinal mechanism. The study points out that interventions aimed at strengthening psychological flexibility are crucial for fostering students' long‐term career development.

### 5.2 Theoretical Implications

The study's findings yield significant theoretical implications that expand present understanding of ACT, CCT, and the CCMA. Through the empirical convergence of various frameworks, the study offers substantial evidence for the long‐lasting effect of psychological flexibility on future‐oriented career behaviors.

The findings connect ACT and CCT by identifying psychological flexibility as an essential factor of “adaptive readiness.” Although ACT describes psychological flexibility as a crucial regulatory mechanism for responding to environmental demands (Hayes et al. 1996, 2011), previous research has mostly concentrated on this concept in the context of general well‐being rather than vocational development (Waldeck et al. 2021; Russo et al. 2024). This study enriches the literature by revealing that psychological flexibility acts as a crucial competency that activates career adaptability resources. It was previously described as a capability for behavioral regulation and opportunity recognition (Bond et al. 2008; Hayes et al. 2011). In accordance with Waldeck et al. (2021) and Kirikkanat (2023), the results show that those with psychological flexibility are more effective at adapting their cognitions and expectations, thereby developing the psychosocial resources essential for career adaptation. This applies ACT concepts to student career development, highlighting that flexible behavioral control is vital for promoting career‐specific adaptability during education. This also expands the taxonomy of adaptive readiness within CCT, positioning psychological flexibility as a theoretically important dispositional resource that continuously prepares individuals to engage with the adaptability system.

The study provides direct empirical evidence for the sequential mechanism posited by the CCMA (Savickas 2013). The theory proposes a framework wherein adaptive readiness (psychological flexibility) enhances adaptability resources (career adaptability), leading to adaptive responses (proactive career behaviors). This sequence is often theorized, but empirical evidence has primarily depended on cross‐sectional methods (Nilforooshan and Salimi 2016; Taber and Blankemeyer 2015). This research utilizes a longitudinal design to establish causality, demonstrating that psychological flexibility acts as the catalyst for the overtime accumulation of adaptability resources (Hayes et al. 2011).

The mediating function of career adaptability in the association between psychological flexibility and proactive career behaviors has been inadequately explored in the literature. Research indicates that career adaptability serves as a significant mediating variable (Wang, Li, et al. 2024; Russo et al. 2024). The study supports the mediating function of career adaptability, addressing the theoretical gap between personal attributes and behavioral implications. Prior studies have often regarded career adaptability merely as flexibility or a driver to proactivity (Nilforooshan and Salimi 2016; Wang, Li, et al. 2024). Also, previous studies centered on psychological flexibility as a predictor of overall well‐being (Bond et al. 2010; Masuda and Tully 2012) or on career adaptability as a driver of career outcomes (Hirschi et al. 2015; Urbanaviciute et al. 2016). This study establishes that career adaptability acts as a unique mechanism that converts psychological flexibility into proactive career behaviors, thereby affirming that it acts as a “resource” that drives goal‐oriented actions (Hirschi et al. 2015). This shows that proactive career behaviors are not solely the result of personality traits but rather the result of a resource‐development process driven by psychological flexibility. Moreover, the result shows how psychologically flexible individuals convert their internal regulatory abilities into exploratory, self‐directed career behaviors. This signifies a theoretical progression by clarifying the convergence of ACT's notion of flexible behavioral control with CCT's framework of career adaptability.

### 5.3 Practical Implications

This study offers some tangible, practical, and administrative implications for human resources professionals, career centers, career consultants, and higher education institutions.

The study demonstrates that university career centers should adopt a two‐stage psychoeducational curriculum that prioritizes developing students' internal resources above technical abilities, going beyond traditional CV writing workshops. Students must learn how to manage career‐related anxiety before they can begin career planning. Therefore, the first stage should employ ACT techniques in terms of adaptive readiness. This intervention technique ensures that students initially develop the requisite adaptive readiness (flexibility) to proficiently employ adaptability resources (planning and exploration). Cognitive defusion and acceptance strategies can be effectively included in career planning courses or workshops to assist students in addressing career‐related anxiety. For instance, students can be instructed to recognize upsetting beliefs like “I will never find a job” without accepting them as definitive realities. By cultivating this awareness, students can distinguish themselves from habitual negative assessments, thereby diminishing experiential avoidance and encouraging more effective coping methods in their career planning and decision‐making processes. Present‐moment awareness can be fostered through mindfulness practices, which may be incorporated into academic curricula to assist students in concentrating on their ongoing skill enhancement. By promoting focus on the present instead of dwelling on ambiguous future outcomes, these strategies assist students in alleviating anxiety associated with job uncertainty and augmenting their involvement in proactive learning and career planning endeavors. Upon the enhancement of psychological flexibility, the second stage must concentrate on the competencies of CCT. Fostering exploration and curiosity in students can be provided by offering low‐stakes employment experiences, such as job shadowing and informational interviews. Students possessing greater psychological flexibility might especially gain from these activities, since they enable the application of their openness to tangible career exploration. Through participating in these experiences, students can convert abstract curiosity into practical insights, thereby deepening their comprehension of various career paths and guiding their future career choices. Control and confidence can be developed through organized group coaching sessions that assist students in transforming their curiosity into practical decision‐making abilities and self‐efficacy. Through participation in guided conversations, reflective exercises, and collaborative problem‐solving, students cultivate a greater feeling of agency regarding their career decisions and an augmented belief in their capacity to effectively address career obstacles. These strategies facilitate proactive professional actions and improve overall career readiness.

The finding of career adaptability substantially predicted proactive career behaviors suggests to career counselors that improving adaptation resources will enhance students' career participation in the long term. As a result, university career centers might include focused coaching programs and systematic career adaptation evaluations in their offerings. Interventions involving individual and group coaching that prioritize autonomy in decision‐making, future planning, and exploratory skills can assist students in developing the flexibility required to successfully enter the workforce. Such interventions should encourage more initiative and participation in career‐related tasks, which is in line with previous research that suggests psychological resources shape proactive behavior (Nilforooshan and Salimi 2016; Urbanaviciute et al. 2016).

Human resource professionals aiming to develop a proactive early‐career workforce may use psychological flexibility as a significant selection criterion. Recruitment methods can be improved by integrating validated tests of psychological flexibility, such as the Acceptance and Action Questionnaire‐II (AAQ‐II), with conventional technical evaluations. The study indicates that individuals exhibiting greater psychological flexibility are more inclined to cultivate adaptability resources and participate in proactive behaviors over time, as also indicated by a cross‐sectional study (Kuo et al. 2018). By recognizing and selecting individuals possessing these attributes, organizations can develop a workforce more effective at managing uncertainty and responding effectively to dynamic and flexible work settings. Job crafting interventions, in which managers encourage new employees to adapt to certain areas of their jobs to better align with their personal values and skills, can improve onboarding processes. In addition to promoting personal fulfillment and engagement, these practices strengthen the connection between proactive behavior and psychological adaptability. Organizations cultivate a workforce that can independently adjust to changing job demands and dynamic market conditions by allowing employees to meaningfully shape their roles.

### 5.4 Limitations and Future Research

Scientific studies may have some limitations due to their nature. This study possesses certain limitations. Consequently, all identified research constraints must be scrutinized and considered when assessing the results. A primary and significant limitation of the conducted research is the sample. The research data were gathered by a convenience sample method from Turkish‐origin university students enrolled at a state university in Türkiye. Although the university accommodates a broad student body with its high student numbers, dependence on a singular institution may restrict the applicability of the findings to students from other cultural or institutional backgrounds. Subsequent research should utilize multi‐center sampling methodologies to enhance the validation of the model. Therefore, it is advisable to interpret the results while considering the developmental traits, cultural specifics, age, and gender of the participants. Furthermore, future cross‐cultural studies may be undertaken by collecting data from individuals with diverse traits. This variable has been thoroughly examined within Western culture; however, cross‐cultural studies can elucidate unique contexts pertinent to different cultures and uncover the mechanisms underlying the establishment and evolution of proactive career behaviors in that context. Also, the measurement instruments employed in this study rely on self‐reporting, which is regarded as a drawback of the research. Data may be acquired by alternative methodologies in forthcoming scientific investigations.

The final restriction of the present research is that, despite the study's longitudinal nature, a significant methodological restriction is the use of only two assessment points. While assessing temporal precedence is possible with two‐wave designs, they provide little information about intra‐individual change and developmental trajectories (Little 2013). The study used a month's gap between measurement waves to effectively analyze short‐term relationships among variables. To more thoroughly capture the changes and phases of development of important attributes such as career adaptability, utilizing extended time periods such as 6–12 months may be ideal. It is advisable to implement longer‐term longitudinal procedures in forthcoming studies. Future studies should improve both the quantity and frequency of measurements to more effectively elucidate and assess the correlations among these variables. A comparable pattern can be examined using a fully cross‐lagged panel design with three, four, or additional data sets. This approach allows for a clearer statistical determination of the relationships between variables and their impact on one another.

## 6 Conclusion

This longitudinal study identified that students' career adaptability mediates the association between their psychological flexibility and proactive career behaviors. Emphasizing methods that enhance individuals' psychological flexibility is beneficial for fostering proactive career behaviors and delivering services that facilitate this development. Furthermore, students may be guided toward activities that enhance their career adaptability. Consequently, their proactive career behavior portfolio may evolve.

## Funding

The authors have nothing to report.

## Ethics Statement

Ethical permission was obtained from the Ethical Compliance Review Board for Scientific Research of Çukurova University Adana/Türkiye (30.11.2022‐E.569391).

## Conflicts of Interest

The authors declare no conflicts of interest.

## Data Availability

The data that support the findings of this study are available on request from the corresponding author. The data are not publicly available due to privacy or ethical restrictions.
